# VEGF +936 C/T Genetic Polymorphism in Patients with Cervical Dysplasia

**DOI:** 10.1155/2016/6074275

**Published:** 2016-10-12

**Authors:** Ioana Cristina Rotar, Diana Elena Dumitras, Radu Anghel Popp, Felicia Maria Petrisor, Paul Cotutiu, Florin Stamatian, Daniel Muresan

**Affiliations:** ^1^1st Clinic of Obstetrics and Gynecology, University of Medicine and Pharmacy “Iuliu Hatieganu”, Clinicilor 3-5, 400006 Cluj-Napoca, Romania; ^2^Department of Economic Sciences, University of Agricultural Sciences and Veterinary Medicine Cluj-Napoca, Calea Manastur 3-5, 400372 Cluj-Napoca, Romania; ^3^Department of Molecular Sciences, University of Medicine and Pharmacy “Iuliu Hatieganu”, Clinicilor 3-5, 400006 Cluj-Napoca, Romania; ^4^1st Clinic of Obstetrics and Gynecology, Emergency County Hospital, Cluj-Napoca, Romania

## Abstract

*Aim*. The present study aims to analyze the potential role of VEGF +936 C/T polymorphism in cervical intraepithelial neoplasia.* Material and Method*. One hundred and eighty-six patients were included in the study: 75 cases (patients diagnosed with CIN) and 111 controls (negative for both HPV testing and cytology). For each patient a single visit was scheduled when colposcopy was performed. From cervical specimen, cytology and HPV testing were performed and from peripheral blood VEGF +936 genotyping was determined. For statistical analysis purposes OR and chi-square were used at a level of significance of <0.05.* Results*. No link has been found in the detection of CT genotype in cases versus controls, OR = 0.8295, [0.42, 1.62]. An inverse correlation has been found between T allele and HSIL, OR = 0.2121, [0.0473, 0.9517], *p* = 0.0866.* Conclusion*. No link has been found between VEGF +936 C/T and cervical intraepithelial neoplasia.

## 1. Introduction

Cervical cancer represents even nowadays an important factor of morbidity and mortality being considered as the fourth most frequent cancer in women worldwide [1]. Cervical intraepithelial neoplasia corresponds to premalignant lesions [2, 3]. WHO estimates that about 1-2% of all women are diagnosed every year with a CIN+ (cervical intraepithelial neoplasia) lesion every year [4]. HPV plays a pivotal role in cervical carcinogenesis [5, 6].

Squamous cervical cancer is imagined as a continuous process that takes place above the basal membrane of the cervical squamous epithelium [2, 6]. Different classes of cervical intraepithelial neoplasia LSIL (low grade intraepithelial lesion) and HSIL (high grade intraepithelial lesion) corresponding to the bioptic CIN I and, respectively, CINs II and III are seen as different stages of the same condition that can either progress or regress to another cytological category [2]. In LSIL, HPV HR is in most of the patients located in the cytoplasm in an episomal state [2]. In case of persistent HPV HR (High Risk Human Papilloma Virus) infections the formal double circular HPV is disrupted and later integrated in the nuclear host cell DNA leading to the secretion of large amounts of E6 and E7 viral oncoproteins [2].

VEGF (vascular endothelial growth factor) represents a family of polypeptides including VEGF-A, VEGF-B, VEGF-C, VEGF-D, VEGF-E, and placental growth factor (PLGF) [7]. VEGF-A gene is located on 6p21.3 and has eight exons [8]. VEGF acts upon VEGF receptors promoting arterial, venous, and lymphatic vasculogenesis [9]. Moreover VEGF plays essential roles in tumor growth and metastasis [10].

The upregulation of E6 and E7 HPV oncoproteins inactivates tumors repressors p53 and Rb (retinoblastoma) interfering with cell-cycle control, key step of cervical carcinogenesis [11]. Also in primary foreskin keratinocytes the expression of the same molecules, HPV 16 E6 and E7, was associated with increased amounts of VEGF (vascular endothelial growth factor) [12]. Moreover VEGF levels have been found to be elevated in cervical cancers and its precursors [13].

Neoangiogenesis is present in early stages of cervical neoplasia CINs (cervical intraepithelial neoplasia). Vascular pattern assessment represents an important step of colposcopy that helps to stratify the intraepithelial lesions. Vessels are best visualized by using a green filter. Abnormal vessels depicted as mosaic, punctuation, pollarded vessels depending on their characteristic (broadness, appearance, pattern, and branching) are commonly seen in intraepithelial neoplasia and microinvasive cervical cancer [14].

VEGF exerts important effects upon cervical angiogenesis; therefore, different VEGF genotypes may have impact upon cervical carcinogenesis. The aim of the present study is to analyze the relationship between VEGF +936 C/T (rs3025039) genotypes and cervical intraepithelial neoplasia.

## 2. Materials and Methods

A total number of 186 patients were enrolled in the present study at 1st Clinic of Obstetrics and Gynecology, Cluj-Napoca, Romania, in 2014. The research was designed as a prospective case control including 75 cases and 111 controls. Inclusions criteria for cases were represented by cytological diagnosis of CINs. The controls were represented by age-matched females with a normal cytology and negative HPV. The refusal to participate in the study was a firm exclusion criterion. After signing the informed consent form all patients were scheduled for a clinical visit. The specific study form was filled for each patient including anamnestic data (age, personal history, obstetric history, and previous CIN). Every patient had a clinical examination followed by colposcopy respecting the four steps (native, green filter, acetic acid, and Lugol's Iodine solution) using a digital video colposcope (MIKRO MZ6, Prague, Czech Republic) with integrated camera Leica IC80HD® (Prague, Czech Republic). For a better assessment of vascular pattern the green filter was used whenever necessary also after acetic acid application. A cervical specimen was obtained for each patient before the colposcopic examination. In cases of clinical indication a specimen biopsy was taken under colposcopic guidance. All patients with HSIL (high grade squamous intraepithelial lesion) were referred for cold knife conization. At the end of the visit 2 mL of peripheral blood was drawn.

Cervical specimens were used for performing liquid based cytology and HR-HPV typing. The cervical cytology was examined in the laboratory of the above mentioned university hospital according to the Bethesda nomenclature [15].

The DNA extraction and genotyping was performed at the Department of Medical Genetics. Genomic DNA was extracted from 300 *μ*L peripheral blood samples using Wizard Genomic DNA Purification Kit (Wizard® Genomic DNA Purification Kit, Promega, MA, USA). The DNA was stored at −20°C before genotyping. VEGF +936 C/T SNP (single nucleotide polymorphism) rs3025039 was detected using a RFLP (restriction fragment length polymorphism) as described previously [16].

The study database was created and later analyzed using Microsoft Excel 2007® and STATA Intercooled 10® (College Station, Texas, USA). *t*-test was used for comparison of different classes of women. The relationship between VEGF alleles and genotypes with cervical intraepithelial neoplasia was assessed using the chi-square test and the odds ratios (OR) with 95% confidence interval (CI).

## 3. Results

A total number of 186 patients were enrolled in the study including 75 cases and 111 controls. The distribution of patients after their respective cytological category is depicted in Table 1. The average age was 40.36 years for cases and 42.32 for controls, respectively. No statistical difference regarding average age has been found between cases and controls (*t* = 1.178, *p* = 0.2404). Moreover no statistical significance was found in the age of the following groups: LSIL/HSIL (*t* = 0.182, *p* = 0.8568), HSIL/CIS (*t* = 1.867, *p* = 0.0685), and LSIL/HSIL (*t* = 1.074, *p* = 0.297).

As expected wild VEGF +936 CC homozygous genotype was the most frequently encountered (cases: 73.33%; controls: 72.97%) while the heterozygous CT was detected in 26.34%. The VEGF +936 TT homozygous genotype was identified in only one patient with in situ carcinoma; therefore, it was excluded for further statistical analysis. The common variant VEGF allele +936 C was prevalent in cases (86%) and in controls (86.49%). The frequencies of VEGF +936 genotypes and alleles are depicted in Table 2.

While examining the relationship between VEGF +936 heterozygous CT genotype and different CINs categories no statistical significant association has been found (data are showed in Table 3). However, data suggested potential protective effect: cases/controls, OR = 0.8295, CI 95% [0.4229, 1.6271], 0.0521, the result being at the limit of significance. The effect seems even more protective for HSIL (OR = 0.6452, *p* = 0.0667) than for LSIL (OR = 0.7373, *p* = 0.367) but still not significant. The complete analysis of the relationship between VEGF genotype and cervical dysplasia can be found in Table 3.

In Table 4 allelic frequencies are investigated separately in patients with different CINs and controls. No statistical significant differences have been found between cases and controls and, respectively, different CINs and controls. Interestingly the presence of T alleles suggests a significantly different behavior in patients with HSIL than LSIL exerting a potential protective effect (OR = 0.2121, CI 95% [0.0473, 0.9517], *p* = 0.0866).

## 4. Discussion

SNP (single nucleotide polymorphism) could be located in any region of a gene structure including introns or exons. SNPs either can have no effect or could upregulate or downregulate gene expression [17]. They occur with a frequency of 1% of the human population and can influence the response of the human body to environmental factors, diseases, drugs, and therapies [17]. The studied polymorphism is located within the promoter, key region of the gene expression [17]. In the present study the expression level was not assessed, but other researchers had demonstrated that this particular SNP is functional [18].

VEGF represents an important molecule for cervical dysplasia [13, 19, 20]. Moreover VEGF-C was identified as independent predictor of CIN II [19].

The exact mechanisms of VEGF action in cervical carcinogenesis are not fully understood. It is not even known exactly what type of cells secretes VEGF being incriminated tumor cells themselves [10, 21] or tumor stromal cells [10]. Certainly VEGF plays a pivotal role in angiogenesis and critical step in solid tumorigenesis including cervical location [10, 21]. In the presence of persistent HPV HR in the integrated form E6 downregulates p53 that stimulates VEGF synthesis [22]. Moreover HPV E6 independently increases VEGF production by stimulating hypoxia inducible factor 1 alpha (HIF-1 alpha) secretion [23].

VEGF stimulates the formation and differentiation of new vessels within the tumor, extremely important in the process of tumor growth [13, 24]. In cervical cancer patient high levels of VEGF are independent negative predictor factor for radiotherapy [25] and chemotherapy [26]. Moreover Wu et al. [27] had demonstrated that VEGF mRNA from liquid based cytology samples can be a useful tool in the triage of abnormal Papanicolaou results.

VEGF is secreted in high amounts in CINs [13], a positive correlation being demonstrated between VEGF levels and different stages of CINs and of cervical cancers [28–30].

The presence of T allele at VEGF +936 is associated in healthy women with a lower level of secretion [18]. These findings suggested that the presence of CT genotype (in our study we had only one case of TT genotype; therefore, we could not perform a statistical analysis only on the later genotype) is associated with lower VEGF level. Moreover a presumable diminished VEGF expression might exert protective effects for CIN (genotype CT in cases/controls OR 0.8295, CI 95% [0.4229, 1.6271]). The protective effect seems to be progressive from LSIL to HSIL (LSIL, OR = 0.7373; HSIL, OR = 0.6452). The comparisons between cases, here including LSIL and HSIL, and controls did not reach the level of statistical significance, even if the *p* value of the comparison between cases and controls was extremely close to the superior limit (0.0521). Moreover a presumably protective effect is present in patients with HSIL comparative to LSIL OR = 0.2121, [0.0473, 0.9517]. Taking into account all the *p* values from Tables 3 and 4, we cannot conclude that there is link between VEGF +936 CT polymorphism and cervical dysplasia.

Previous studies from the literature had shown that VEGF expression is directly proportional to the CIN levels, cervical cancer stages, and prognosis [28–30]; therefore, an innate potential low VEGF could modulate cervical carcinogenesis.

To date, we have identified only two articles that have analyzed the relationship between cervical cancer and VEGF +936, the results being similar with no statistical significant link found between VEGF +936 genotype and cervical cancer: CT genotype by Kim et al. (2010), cases/controls (*n* = 199/215), OR = 0.88, CI 95% (0.62, 1.23), *p* = 0.45 [31], and, respectively, by Konac et al. (2007), cases/controls (*n* = 32/106), OR = 0.52, CI 95% (0.22–1.23), *p* = 0.133 [32]. The values of OR were similar in the three studies except for one showing potential protective effect.

The relationship between VEGF genetic polymorphism and cervical dysplasia is far from being elucidated in our opinion. The present study opens future research directions; it would be interesting to analyze the colposcopic appearance, VEGF polymorphisms, and VEGF mRNA.

## Figures and Tables

**Table 1:** Patients classification according to the cytological diagnosis.

	Cytological classification of the cervical dysplasia	Number of patients
Controls	NILM	111

Cases	ASC-US	10
	ASC-H	10
	LSIL	9
	HSIL	35
	CIS	11
	LSIL + HSIL + CIS	55
	HSIL + CIS	46
	Total	75

NILM, negative for intraepithelial lesion; ASC-US, atypical squamous cells of undetermined significance; ASC-H, atypical squamous cells—cannot exclude HSIL; CIS, in situ carcinoma.

**Table 2:** The distribution of VEGF +936 genotypes and alleles according to the cytological categories.

Cytological category	Genotypes						Alleles			
	CC		CT		TT		C		T	
	Number	%	Number	%	Number	%	Number	%	Number	%
NILM	81	72.97	30	27.03	0	NA	192	86.49	30	13.51
ASC-US	3	30.00	7	70.00	0	NA	13	65.00	7	35.00
ASC-H	8	80.00	2	20.00	0	NA	18	90.00	2	10.00
LSIL	5	55.56	4	44.44	0	NA	14	77.78	4	22.22
HSIL	31	88.57	4	11.43	0	NA	66	94.29	4	5.71
CIS	8	72.73	2	18.18	1	9.09	18	81.82	4	18.18

NA: not applicable.

**Table 3:** VEGF +936 genotype CT analysis.

Comparison	OR	OR 95% CI	Chi-squared	*p*
Cases/controls	0.8295	(0.4229, 1.6271)	3.774	0.0521
LSIL/controls	0.7373	(0.1451, 3.7455)	0.393	0.5305
HSIL/controls	0.6452	(0.2555, 1.6290)	3.363	0.0667
CIS/controls	0.6000	(0.1225, 2.9376)	0.402	0.5262

**Table 4:** VEGF +936 alleles analysis; T versus C.

Comparison	OR	OR 95% CI	Chi-squared	*p*
Cases/controls	1.0419	(0.5714, 1.8996)	0	0.985
LSIL/controls	1.8286	(0.5642, 5.9268)	0.446	0.5042
HSIL/controls	0.3879	(0.1317, 1.1423)	2.435	0.1186
LSIL + HSIL + CIS/controls	0.7837	(0.3844, 1.5977)	0.032	0.859
HSIL + CIS/controls	0.6095	(0.2682, 1.3853)	0.999	0.3176
HSIL/LSIL	0.2121	(0.0473, 0.9517)	2.937	0.0866
HSIL + CIS/LSIL	0.3333	(0.0884, 1.2565)	0.923	0.3367
